# Response to comment on ”Identifying patterns differing between high-dimensional datasets with generalized contrastive PCA”

**DOI:** 10.1371/journal.pcbi.1013557

**Published:** 2025-10-30

**Authors:** Eliezyer Fermino de Oliveira, Lucas Sjulson

**Affiliations:** 1 Dominick P. Purpura Department of Neuroscience, Albert Einstein College of Medicine, Bronx, New York, United States of America; 2 Department of Psychiatry and Behavioral Sciences, Albert Einstein College of Medicine, Bronx, New York, United States of America; Universitatsklinikum Hamburg-Eppendorf, GERMANY

A recent formal comment published in PLOS Computational Biology highlights the relationship between generalized contrastive PCA (gcPCA) [1] and the framework of generalized eigenvalue decomposition (GED) [2]. We thank Woller et al. [3] for their thoughtful analysis and for drawing our attention to relevant prior literature that we did not cite in the original paper [4–8]. We fully agree with their observation that the procedure used to optimize gcPCA’s objective function is mathematically equivalent to GED, and that this is true of other contrastive methods as well, including Linear Discriminant Analysis (LDA). Accordingly, rather than the statement “gcPCA is equivalent to GED,” it is more precise to say instead that gcPCA belongs to a larger family of GED-based data analysis methods that also includes LDA.

Woller et al. [3] further argued that gcPCA should be regarded as a supervised method because it requires label information to distinguish datasets A and B. While we understand their reasoning, we believe it is important to clarify why gcPCA does not fit the conventional definition of a supervised method. In our view, the defining hallmark of supervised approaches is not the presence of labels, but rather the use of explicit examples of desired outputs to train a model [9–11]. LDA is unequivocally supervised because class labels directly specify the outputs the model is trained to predict. In contrast, gcPCA relies on labels only to define two datasets to be contrasted, but the outputs of the method are not equivalent to those labels. The distinction is critical because gcPCA is easily confused with LDA, and categorizing it as supervised reinforces this confusion. On the other hand, we agree that a distinction must also be drawn between gcPCA and standard PCA, which uses no label information at all. gcPCA occupies a middle ground in which it uses labels to structure the contrast, but its outputs are not label-equivalent predictions. This highlights that the terms “supervised” and “unsupervised” represent an overly rigid dichotomy that poorly describes contrastive dimensionality reduction [10].

We would also like to address a point of confusion regarding the orthogonalization process in orthogonalized gcPCA. In standard PCA, the ordering of components is straightforward: eigenvalues are nonnegative, with the largest positive values first and the values closest to zero last. In gcPCA, eigenvalues can be both positive and negative. Here, the ordering places the largest positive eigenvalues first, the largest negative eigenvalues last, and the eigenvalues closest to zero in the middle. Accordingly, orthogonalized gcPCA alternates between the first and last components since these have the largest eigenvalue magnitudes. We hope this clarification will help readers better understand the basis for the ordering of components in our implementation.

Woller et al. [3] also emphasized the benefits of non-orthogonal components, and we agree that these can be valuable in certain contexts. In our view, the choice between orthogonal and non-orthogonal gcPCs should be guided by the data analysis goal. When the aim is to study the properties of individual components, non-orthogonal gcPCs may be advantageous, as they more faithfully preserve relationships with the original feature space. However, when the objective is dimensionality reduction, orthogonal components are generally preferable because they form an orthogonal basis for a lower-dimensional subspace. For this reason, we provide both options in the gcPCA toolbox and leave the choice to the end user.

In closing, we again thank Woller et al. [3] for their constructive and insightful commentary. Their contribution has clarified the mathematical relationship between gcPCA and GED and also helped position gcPCA within the broader landscape of statistical methods. We hope that this exchange will help researchers more clearly appreciate both the algorithmic foundations and methodological implications of gcPCA. More broadly, we view this dialogue as a valuable step toward refining and extending the use of contrastive approaches in the analysis of high-dimensional datasets.
